# Multiparameter Electromyography Analysis of the Masticatory Muscle Activities in Patients with Brainstem Stroke at Different Head Positions

**DOI:** 10.3389/fneur.2017.00221

**Published:** 2017-05-29

**Authors:** Chuyao Jian, Miaoluan Wei, Jie Luo, Jiayin Lin, Wen Zeng, Weitian Huang, Rong Song

**Affiliations:** ^1^Key Laboratory of Sensing Technology and Biomedical Instrument of Guang Dong Province, Guangdong Provincial Engineering and Technology Center of Advanced and Portable Medical Devices, Sun Yat-sen University, Guangzhou, China; ^2^Department of Stroke Rehabilitation, Guangdong Work Injury Rehabilitation Center, Guangzhou, China

**Keywords:** stroke, entropy, vestibular stimulation, masticatory muscles, median frequency

## Abstract

The performance of the masticatory muscle is frequently affected and presents high heterogeneity poststroke. Surface electromyography (EMG) is widely used to quantify muscle movement patterns. However, only a few studies applied EMG analysis on the research of masticatory muscle activities poststroke, and most of which used single parameter—root mean squares (RMS). The aim of this study was to fully investigate the performance of masticatory muscle at different head positions in healthy subjects and brainstem stroke patients with multiparameter EMG analysis. In this study, 15 healthy subjects and six brainstem stroke patients were recruited to conduct maximum voluntary clenching at five different head positions: upright position, left rotation, right rotation, dorsal flexion, and ventral flexion. The EMG signals of bilateral temporalis anterior and masseter muscles were recorded, and parameters including RMS, median frequency, and fuzzy approximate entropy of the EMG signals were calculated. Two-way analysis of variance (ANOVA) with repeated measures and Bonferroni *post hoc* test were used to evaluate the effects of muscle and head position on EMG parameters in the healthy group, and the non-parametric Wilcoxon signed rank test was conducted in the patient group. The Welch–Satterthwaite *t*-test was used to compare the between-subject difference. We found a significant effect of subject and muscles but no significant effect of head positions, and the masticatory muscles of patients after brainstem stroke performed significantly different from healthy subjects. Multiparameter EMG analysis might be an informative tool to investigate the neural activity related movement patterns of the deficient masticatory muscles poststroke.

## Introduction

Stroke, a cerebrovascular disease with a high incidence and a high mortality rate, is divided into hemorrhagic and ischemic types (1). The cerebral damage in survived patients was often left with sequela of motor dysfunction and abnormal muscle activation. Patients frequently have disorders of masticatory system, with masticatory muscle activity, bite force, flexibility of tongue, lip force and chewing performance affected, especially after brainstem stroke (2). It is important for stroke patients to go through a rehabilitation training and recover the masticatory function. However, the impairments of the masticatory system are usually of a high heterogeneity, and the effect of the masticatory rehabilitation training should be enhanced. To improve the effect of training, the overall characteristics of the impaired masticatory system and their responses to neural stimuli in patients after stroke should be studied.

The motor nucleus of the trigeminal in the brainstem is responsible for providing motor innervation to the masticatory muscles (3). The vestibular stimuli caused by changing head positions can affect the performance of the masticatory muscles. Evidences shown in the studies of Funakoshi et al. (4) and Deriu et al. (5) proved that the activation of masticatory muscles was different at different head positions. On the other hand, Kushiro et al. (6) showed a reverse effect that chewing gums increased postural stability when people stood uprightly. Besides, animal experiments suggested that there could be an anatomical connection between the vestibular nuclei and trigeminal motoneurons in the brainstem (7–9). We hypothesized that the impairment of the brainstem might interrupt the responses of the masticatory system to the changes in head position. However, former studies were performed in healthy people. Therefore, the effect of changes in head position on the performance of the masticatory muscles needs further studies.

Surface electromyography (EMG) is a useful tool for quantifying the activation patterns of muscles. However, there were only a few studies evaluating the impairment of masticatory system based on an EMG signal analysis. Cruccu et al. (10) and Wang et al. (11) used a linear EMG parameter, root mean squares (RMS), to evaluate the excitability of muscles (12) and found that the activation of the masticatory muscles was lower in the affected side of patients poststroke during clenching. Another linear parameter, median frequency (MDF), is also widely used to describe spectral characteristics of EMG signals with a good specificity and sensitivity in reflecting the muscle electrophysiology (13, 14). Due to the non-linearity and complexity of EMG signals, it has been reported that non-linear EMG parameters should be included (15, 16). Entropy, as a non-linear parameter, is introduced to assess the complexity of the EMG signal. Approximate entropy (ApEn) and sample entropy were two of the most used entropy estimations. Giannasi et al. (17) evaluated the reliability of several EMG parameters in the masticatory muscles of cerebral palsy patients and demonstrated that RMS, MDF, and ApEn were most reliable. Fuzzy approximate entropy (fApEn) uses an exponential fuzzy function to enhance the consistency and monotonicity, and it is considered as an improved version of ApEn (18). Previous studies reported that fApEn was related to motor unit recruiting and firing (19). A combination of above linear and non-linear EMG analysis would help to obtain more information, from different perspectives, about the performance of the masticatory muscles.

This study aimed to investigate whether there existed a certain activation pattern of the masticatory muscles in patients after brainstem stroke. Fifteen healthy subjects and six brainstem stroke patients were recruited and requested to occlude as hard as possible at five different head positions: (1) upright position; (2) turning left by 30° (left rotation); (3) turning right by 30° (right rotation); (4) turning up by 30° (dorsal flexion); (5) turning down by 30° (ventral flexion). In the meanwhile, surface EMG signals of bilateral temporal anterior and masseter muscles were recorded and analyzed with RMS, MDF, and fApEn.

## Materials and Methods

### Subject Recruitment

Six brainstem stroke patients (five males, one female, mean age was 59.33 ± 13.79 years) were recruited from Stroke Rehabilitation Department at Guangdong Work Injury Rehabilitation Center. This study received permission from local ethics committee and volunteered to take part in this study. They all signed the written informed consent about the purpose and procedures of the study prior to the experiment. The neurologist accompanied beside the patients during the experiment to avoid accidents. The clinical diagnosis of the patients was evaluated by neurologist based on MRI or CT scanning images. No patient had a history of neurological disorders or symptoms prior to stroke. The Function Oral Intake Scale (FOIS) was used to evaluate the masticatory function of patients. Low scores indicated weak masticatory function. The exclusion criteria were: associated diseases such as dental problems, missing teeth (between the premolar and molar in the left and right side), temporomandibular disorder, feeling painful during clenching and changing head positions, and under an orthodontic treatment. The demographic and clinical characteristics of all the patients were listed in Table 1. Specially, one of the patients had no FOIS score, but he did not have any facial asymmetry and did a good job in the experiment.

**Table 1:** **Demographic and clinical information of patients**.

No.	Age (years)	Sex	Duration (months)	Type	FOIS	Facial sagging side
1	58	M	32	H	1	R
2	44	M	28	H	5	R
3	64	F	5	I	5	W
4	43	M	28	H	–	W
5	77	M	16	I	3	W
6	70	M	35	H	1	R

*M, male; F, female; H, hemorrhage; I, ischemia; FOIS, Function Oral Intake Scale; –, without the diagnosis of clinical doctor; L, left; R, right; W, without facial asymmetry*.

Fifteen healthy subjects (8 males, 7 females, age: 22 ± 2 years) were recruited from Sun Yat-sen University. All subjects were in general good health and had a normal occlusion without any pathologic changes in the orofacial myofunction, masticatory system, or cervical spine. The inclusion criteria were completeness of natural permanent teeth, i.e., at least 28 teeth, including complete bilateral molar and premolar. Thirteen healthy subjects habitually chewed with their right sides. One of the remaining two subjects usually used the left side to chew, and the other had no habitually chewing side.

### Recording System

Surface EMG signals were recorded with disposable Ag–AgCl bipolar electrodes. Before the placement of the electrodes, the skin beneath the electrodes was cleaned with 70% alcohol. Surface electrodes were positioned on the muscular bellies of bilateral temporal anterior and masseter muscles of subjects, paralleled to muscular fibers with an inter-electrode distance of 20 mm. For the temporal anterior muscle, the electrodes were placed vertically along the anterior margin of the muscle, while for the masseter muscle, at the lower third of the line between the lateral angle of eye and the gonial angle. Subjects were requested to clench to adjust the location of electrodes. The reference electrodes were positioned at the corresponding ipsilateral elbow joint.

The activation of muscles was recorded by a four-channel EMG amplifier, sampled at 1,000 Hz by a 16-bit data acquisition card (DAQ USB-6341, National Instrument Corporation, Austin, TX, USA). The bandwidth of the on-board analog band-pass filter was 10–500 Hz. A program was designed in LabVIEW™ (LabVIEW 2012, National Instruments Corporation, Austin, TX, USA) to store the data.

### Experimental Protocol

**Figure 1:**
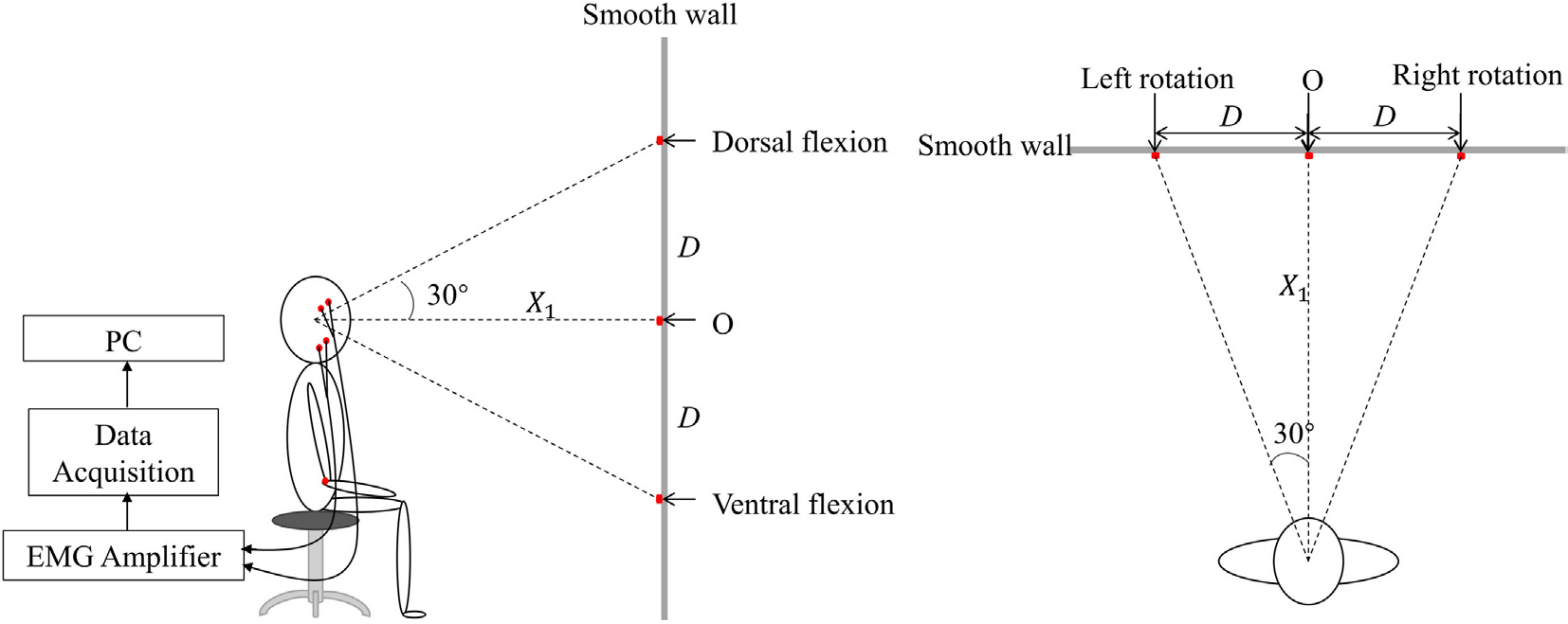
**The lateral and vertical view of the schematic diagram of experiment in the experiment**. There were five markers on the smooth wall. Point O was of the same height as those of the subject’s eyes. The others were, respectively, located on the wall to the left, right, upside, and downside of the point O with a distance *D*, represented various head positions, i.e., left rotation, right rotation, dorsal flexion, and ventral flexion.

Figure 1 described the schematic diagram of the experimental setup. During the experiment, subjects seated straightly on a fixed chair with their hands put on the knees. The knee joints were kept at 90°. A smooth wall, with five markers indicating the five head positions, was in front of the subjects with a distance *X*_1_. Point O on the smooth wall represented the upright position. When the subjects turned their heads to straightly face the other four points, they actually turn their head 30° in the four directions: left, right, up, and down. The height of point O was determined by the height of the participant’s eyes. *X*_1_ was measured before the experiment. The positions of the other four points on the wall representing head positions of left rotation, right rotation, dorsal flexion, and ventral flexion were determined by $D=X_{1}\times \text{tan}30^{{}^{\circ}}$.

**Figure 2:**
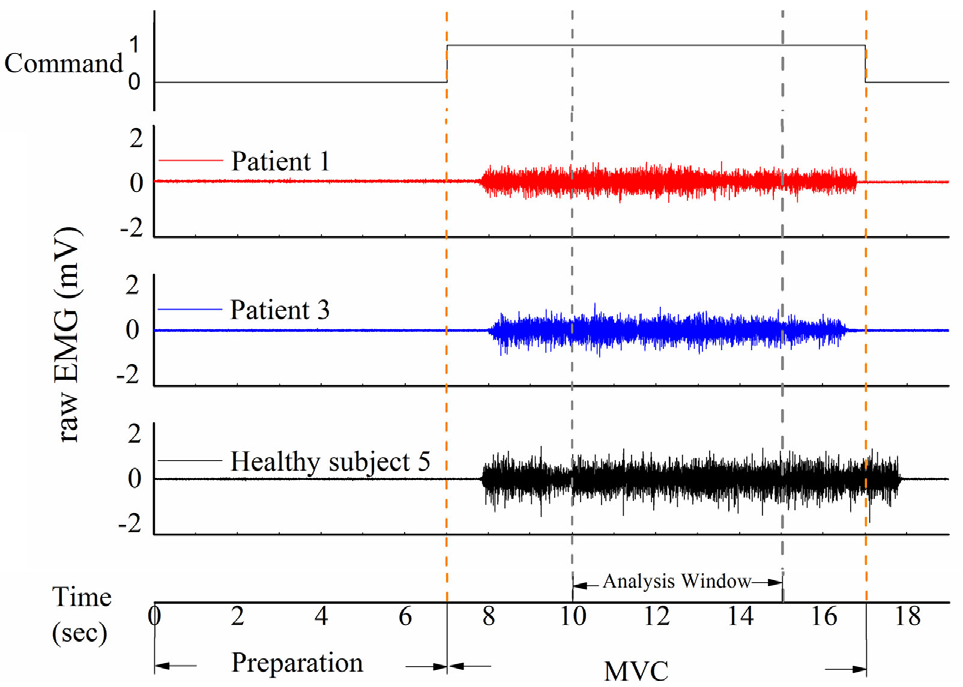
**Three samples of raw electromyography (EMG) signal of the masticatory muscle in response to the command in patients and healthy subjects**. The task was divided into a 7-s preparation phase and a 10-s maximal voluntary clenching (MVC) phase. An analysis window was added from 10 to 15 s.

A short training was conducted before the experiment. Then the subjects were requested to turn their head to straightly face the five different markers on the wall in turn to induce static vestibular stimuli. After each change in head position, the subjects followed the verbal instruction of beginning the clench at 7 s (Figure 2, command), such as “clench,” and they occluded as hard as possible [to generate maximum voluntary clenching: maximal voluntary clenching (MVC)]. The occlusion phase of a trial lasted for 10 s. At each head position, the trial was repeated four times, so the task consisted of five 4-trial blocks. To avoid fatigue, there was a 30-s rest after each trial, and a 2-min rest between each two blocks.

### Data Analysis

A representative example of raw EMG signals of the left masseter muscle of three subjects during MVC in the upright position was displayed in Figure 2. Patient 1 could not eat with mouth and was tube dependent, and his FOIS score was 1. Patient 2 had a FOIS score of 5. Although the two patients had different FOIS scores, their raw EMG signals were similar. The delay between the clenching command and the actual EMG clenching onset was due to the response time of the research assistant and the subject. The duration of the MVC phase was 10 s in each trial, and a 5-s analysis window was added from 3 to 8 s of the clench phase (Figure 2). The raw data were preprocessed with a fourth-order 10–300 Hz band-pass digital Butterworth filter. RMS, MDF and fApEn were calculated using the filtered EMG signals.

Root mean squares represented the amplitude of the signals, and it was calculated using the following formula:

$$\text{RMS}=\sqrt{\frac{1}{N}\sum_{i}u_{i}^{2}}$$

where *u_i_* was the filtered EMG signals (*i* = 0, 1, …, *N* − 1), and *N* was the length of the EMG signals. MDF was defined as the frequency point that divided the spectrum into two equal parts. To calculate MDF, the following equation should be satisfied.

$$\sum_{j=1}^{\text{MDF}}P_{j}=\sum_{j=\text{MDF}}^{M}P_{j}=\frac{1}{2}\sum_{j=1}^{M}P_{j}$$

where *P_j_* represents the power spectrum of the filtered EMG, *j* represents the *j*-th discrete frequency, and *M* represents the bandwidth of the power spectrum analysis.

The calculation steps of fApEn were as follows:

Xim={u(i), u(i+1), …, u(i+m−1)}−u0(i), i =1,2,…,N−m+1, n=N−m+1

where $X_{i}^{m}$ represented an *m* dimensional vector reconstructed with the filtered EMG signals *u*(*i*). *u*0 (*i*) was the average value of the *m* discrete filtered EMG data, and was defined as $u0(i)=\frac{1}{m}\sum_{j=0}^{m-1}u(i+j)$. The distance between two different *m* dimensional vectors was calculated as

$$d_{ij}^{m}={\text{max}}_{i\neq j}\left|X_{i}^{m}-X_{j}^{m}\right|$$

where $X_{i}^{m}$ and $X_{j}^{m}$ were the two reconstructed *m* dimensional vectors, and $d_{ij}^{m}$ was the distance between $X_{i}^{m}$ and $X_{j}^{m}$. According to the concept of fuzzy entropy (18), the smaller the distance, the higher the similarity. The similarity degree of $X_{i}^{m}$ and $X_{j}^{m}$ was determined by a fuzzy function of $d_{ij}^{m}$, *n* and *r*:

$$D_{ij}^{m}\left(n,r\right)=\text{exp}\left(-{\left(\frac{d_{ij}^{m}}{n}\right)}^{r}\right)$$

where $D_{ij}^{m}$ denoted the similarity degree, *r* was the similarity tolerance should be predefined, the parameter *n* reweighted the contribution of distance $d_{ij}^{m}$, e.g., a larger *n* indicating more contribution of smaller distances of $d_{ij}^{m}$.

$$\emptyset^{m}\left(n,\;r\right)=\frac{1}{n-m}\sum_{i=1}^{N-m}\left(\frac{1}{N-m}\sum_{j=1,j\neq i}^{N-m}D_{ij}^{m}\right)$$

where $\emptyset^{m}(n,\;r)$ was averaged similarity. According to above equations, $\left\{X_{i}^{m+1}\right\}$ and $\emptyset^{m+1}$ could be calculated in the same manner, and

$$\text{fApEn(}m,\;n,\;r,\;N\text{)}=\text{ln}\emptyset^{m}(n,\;r)-\text{ln}\emptyset^{m+1}(n,\;r)$$

where fApEn was the fuzzy approximate entropy of an *N* sample time series.

It was important to set proper values of *m, r*, and *n* before calculating the fApEn. Previous studies recommended *m* to use 2 or 3 because the length of the physiological signal was often not long enough to satisfy the need of the lager value of *m* (18), and *m* = 2 was the choice in this study. The similarity boundary was determined by the value of *r* and *n*. A too narrow boundary increased the sensitivity to noise, while a too wide one resulted in information loss. Besides, Sun et al. used the EMG data of a stroke patient to test how the fApEn changed with the values of *N* and *r*, and they found that when *N* > 300 and *r* ranged from 0.02 to 1, there was no crossover effect in the performance of the fApEn (19). Consequently, *N, r*, and *n* were respectively set to 1,000, 0.15, and 2 in this study (18–20).

All above calculations were processed in Matlab (Matlab R2014a, MathWorks Inc., Natick, MA, USA).

### Statistical Analysis

All the parameters were described as mean ± SD in the paper. The level of significance was set at 0.05 (*P* < 0.05). Levene test was used to test for normal distribution of the data. If the Levene test result showed no significant deviations from variance homogeneity, the data were analyzed by three-way analysis of variance (ANOVA) with repeated measures. The three factors were: subject (healthy and patient), muscle (left temporal, right temporal, left masseter, right masseter) and head position (upright position, left rotation, right rotation, ventral flexion, dorsal flexion). If a large variance with non-normal distribution in the data of a subject group, a non-parametric test should be selected to analyze the data (21). Song et al. (22) suggested that the Wilcoxon signed rank test should be applied to analyze the statistical significance in a small sample size of subjects with high heterogeneity. In the current study, the sample size of the patient group was small. It is complex to perform a non-parametric test of multi-factors and their interactions (23, 24). If the patients’ data did not pass the Levene test and did not display normality, we would use the Wilcoxon signed rank test to analyze the within-subject effects of muscle, head position and their interaction in the patient group based on manually paired data. In addition, if one of the effect was non-significant, we merged the data in the corresponding groups and performed the Wilcoxon signed rank test again to perform multiple comparison. If the healthy group passed the Levene test, a two-way analysis of variance (ANOVA) with repeated measures was used to evaluate the influences of the two within-subject factors. Bonferroni *post hoc* test was used to detect the subgroup differences after the ANOVA comparison. To compare the between-subject differences with unequal-variance data, the Welch–Satterthwaite *t*-test was applied and the significant within-subject effect was controlled. All the statistical procedures were computed using the Statistical for Social Science (SPSS) version 22.0.

## Results

**Figure 3:**
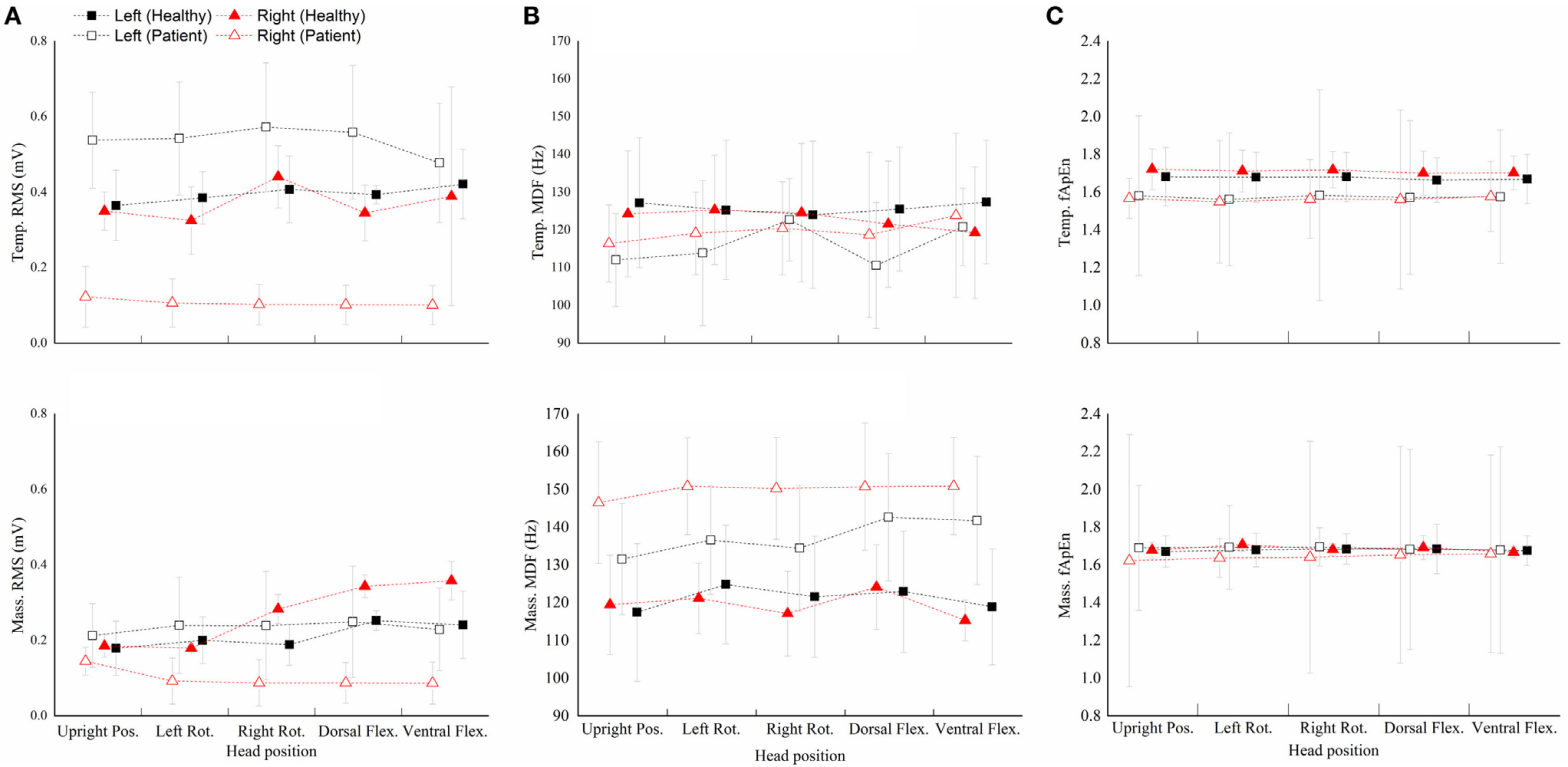
**The root mean squares (RMS) (A), median frequency (MDF) (B), fuzzy approximate entropy (C) means and SDs of the four masticatory muscles at different head positions in healthy subjects and patients**. Temp., temporal anterior muscle; Mass., masseter muscle; Pos., position; Rot., rotation; Flex., flexion.

Figure 3 shows performance of the four masticatory muscles, in terms of RMS, MDF and fApEn, at different head positions. Some trends in the activities of masticatory muscles could be observed in the healthy subjects. For example, when they turned their head to the left, the muscles in the left was activated more than the contralateral ones’, while the muscles in the right had higher RMS means when turning to the right. However, no such trend was observed in the patients. Besides, fApEn means in the patient group had a much larger SD, which surpassed the effect of head position.

The healthy subject’s data passed the Levene test (RMS: *P* = 0.367; MDF: *P* = 0.563; fApEn: *P* = 0.667), but the patients’ data did not pass the Levene test (RMS: *P* = 0.001; MDF: *P* = 0.013; fApEn: *P* < 0.001).

The two-way repeated measure ANOVA results showed that muscle had significant effect (RMS: *P* = 0.001; MDF: *P* = 0.416; fApEn: *P* = 0.179), but head position and muscle × head position interaction had no significant effect (head position: *P* = 0.905; interaction: *P* = 0.818). Bonferroni *post hoc* test showed that the RMS mean of the left masseter muscle was significantly lower than all the other muscles.

**Figure 4:**
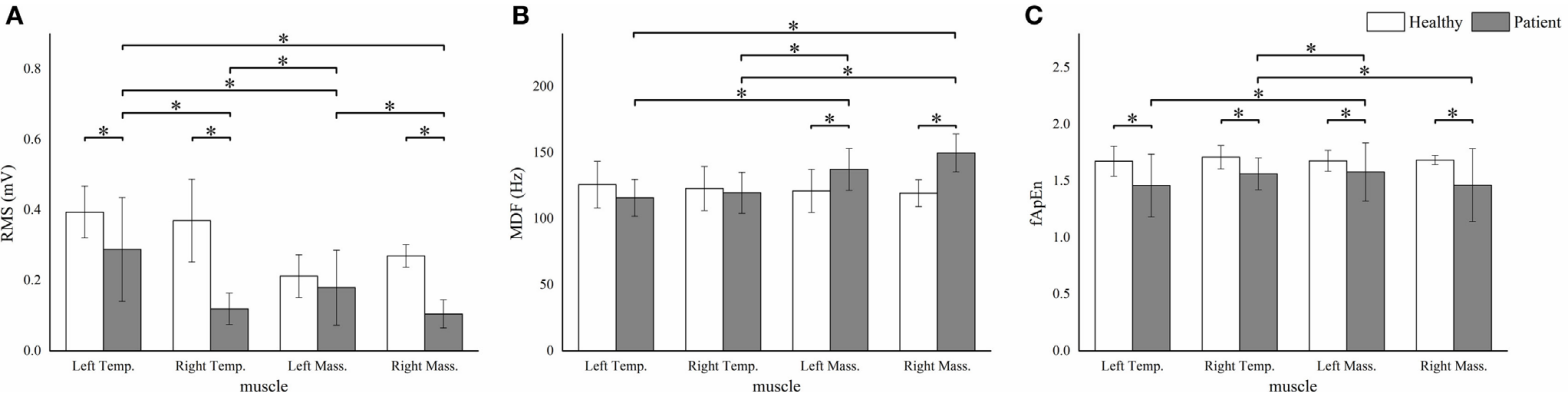
**The root mean squares (RMS) (A), median frequency (MDF) (B), fuzzy approximate entropy (fApEn) (C) means and SDs of the four masticatory muscles in healthy subjects and patients**. *P < 0.05; Temp., temporal anterior muscle; Mass., masseter muscle.

The Wilcoxon signed rank test conducted in the patient group showed a significant effect of Muscle but no significant effect of Head position (data not shown) and muscle × head position interaction (Table 2). Besides, the Wilcoxon signed rank test (Figure 4) indicated that the *RMS* means of any paired muscles were significantly different (*P* < 0.007) except that of the right masseter and temporal muscles, the scores of bilateral masseter muscles were significantly higher than those of bilateral temporal muscles in MDF (*P* < 0.005), and the masseter muscles had significantly higher fApEn means than the ipsilateral temporal muscles (*P* < 0.005).

**Table 2:** **The result of the Wilcoxon signed rank test for statistical significance of muscle × head position interaction on the three electromyography parameters**.

Muscles	Head positions^i^	Head positions^j^	Root mean squares		Median frequency		Fuzzy approximate entropy	
			*Z*	*P*	*Z*	*P*	*Z*	*P*
Left Temp.	Upright Pos.	Left Rot.	−0.447	0.655	−0.447	0.655	−1.342	0.180
		Right Rot.	−0.447	0.655	−1.342	0.180	−0.447	0.655
		Dorsal Flex.	−0.447	0.655	−0.447	0.655	−1.342	0.180
		Ventral Flex.	−1.342	0.180	−1.342	0.180	−1.342	0.180
	
	Left Rot.	Right Rot.	−0.447	0.655	−1.342	0.180	−1.342	0.180
		Dorsal Flex.	−0.447	0.655	−0.447	0.655	−1.342	0.180
		Ventral Flex.	−1.342	0.180	−1.342	0.180	−1.342	0.180
	
	Right Rot.	Dorsal Flex.	−0.447	0.655	−1.342	0.180	−1.342	0.180
		Ventral Flex.	−0.447	0.655	−1.342	0.180	−0.447	0.655
	
	Dorsal Flex.	Ventral Flex.	−0.447	0.655	−1.342	0.180	−1.342	0.180

Right Temp.	Upright Pos.	Left Rot.	−1.342	0.180	−0.447	0.655	−1.342	0.180
		Right Rot.	−1.342	0.180	−0.447	0.655	−0.447	0.655
		Dorsal Flex.	−1.342	0.180	−0.447	0.655	−0.447	0.655
		Ventral Flex.	−1.342	0.180	−0.447	0.655	−1.342	0.180
	
	Left Rot.	Right Rot.	−0.447	0.655	−0.447	0.655	−0.447	0.655
		Dorsal Flex.	−0.447	0.655	−0.447	0.655	−0.447	0.655
		Ventral Flex.	−0.447	0.655	−0.447	0.655	−1.342	0.180
	
	Right Rot.	Dorsal Flex.	−0.447	0.655	−0.447	0.655	−0.447	0.655
		Ventral Flex.	−0.447	0.655	−0.447	0.655	−0.447	0.655
	
	Dorsal Flex.	Ventral Flex.	−1.342	0.180	−1.342	0.180	−0.447	0.655

Left Mass.	Upright Pos.	Left Rot.	−0.447	0.655	−1.000	0.317	−1.342	0.180
		Right Rot.	−0.447	0.655	−0.447	0.655	−0.447	0.655
		Dorsal Flex.	−1.342	0.180	−0.447	0.655	−0.447	0.655
		Ventral Flex.	−1.342	0.180	−1.342	0.180	−1.342	0.180
	
	Left Rot.	Right Rot.	−0.447	0.655	−1.342	0.180	−0.447	0.655
		Dorsal Flex.	−1.342	0.180	−1.000	0.317	−0.447	0.655
		Ventral Flex.	−1.342	0.180	−0.447	0.655	−1.342	0.180
	
	Right Rot.	Dorsal Flex.	−1.342	0.180	−1.342	0.180	−0.447	0.655
		Ventral Flex.	−1.342	0.180	−1.342	0.180	−0.447	0.655
	
	Dorsal Flex.	Ventral Flex.	−0.447	0.655	−0.447	0.655	−0.447	0.655

Right Mass.	Upright Pos.	Left Rot.	−1.342	0.180	−1.000	0.317	−1.342	0.180
		Right Rot.	−1.342	0.180	−0.447	0.655	−1.342	0.180
		Dorsal Flex.	−1.342	0.180	−0.447	0.655	−1.342	0.180
		Ventral Flex.	−1.342	0.180	−1.342	0.180	−1.342	0.180
	
	Left Rot.	Right Rot.	−1.342	0.180	−1.342	0.180	−0.447	0.655
		Dorsal Flex.	−1.342	0.180	−1.000	0.317	−0.447	0.655
		Ventral Flex.	−1.342	0.180	−0.447	0.655	−0.447	0.655
	
	Right Rot.	Dorsal Flex.	−0.447	0.655	−1.342	0.180	−1.342	0.180
		Ventral Flex.	−0.447	0.655	−1.342	0.180	−1.342	0.180
	Dorsal Flex.	Ventral Flex.	−0.447	0.655	−0.447	0.655	−1.342	0.180

*Head positions^i^: one of the head positions in the task; Head positions^j^: a different head position which was used to pair with Head positions^i^; Temp., temporal anterior muscle; Mass., masseter muscle; Pos., position; Rot., rotation; Flex., flexion*.

We observed significant between-subject *RMS* differences in all muscles except the left masseter muscle (*P* < 0.001). The patient group showed significantly higher *MDF* means in the bilateral masseter muscles (*P* < 0.001). Besides, significant lower *fApEn* means were detected in all the muscles except the left masseter muscle (*P* < 0.005) (Figure 4).

**Figure 5:**
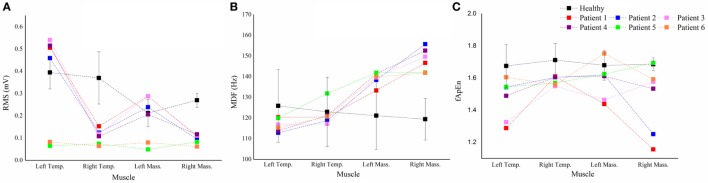
**The case-by-case plot**. Comparison of the root mean squares (RMS) **(A)**, median frequency (MDF) **(B)**, fuzzy approximate entropy (fApEn) **(C)** means of each muscle in each patient with the subject means of the corresponding muscle in all healthy subjects. Temp., temporal anterior muscle; Mass., masseter muscle.

For each parameter, we plotted a case-by-case plot to compare each patient’s mean value with the subject mean in the healthy group (Figure 5). We found that the *RMS* means of Patients 1–4 and Patients 5 and 6 clustered into two patterns: Type I and Type II. Type I was characterized by significantly less activation in the right masticatory muscles, while Type II was characterized by equal weakness of all the four muscles. For the MDF, we found that all the patients’ data varied in the same way, and the mean values of the bilateral masseter muscles laid above the healthy subjects’ corresponding means. The fApEn case-by-case plot showed a large heterogeneity of patients.

## Discussion

This study was designed to investigate the activation pattern of masticatory muscle at different head positions in the healthy subjects and brainstem stroke patients when performing the maximal voluntary clenching (MVC). RMS, MDF and fApEn were used to analyze the EMG data. Significant differences between the healthy subjects and the patients were found in certain muscles.

### Comparison between the Healthy Subjects and Stroke Patients

The lower RMS means of the stroke patients’ right masticatory muscles compared with those of the healthy subjects’ implied that the muscle excitation of their right muscles was quite low. Wang et al. (11) and Cruccu et al. (10) found that the affected side activated lower during clenching. The reduction of the muscle activation in patients was consistent with previous studies.

Compared to healthy subjects, the MDF means of the bilateral masseter muscles in patients was higher. Since MDF was associated with the conduction velocity in the muscle fiber and the action duration of motor unit (25), a higher MDF mean might suggest a higher conduction velocity and a smaller action duration. The shift of the spectrum toward higher frequencies might implied that, after brainstem stroke, the proportion of fast-twitch motor units that were enrolled in masticatory movement was lower (26). Another possible interpretation of the spectrum shift might be the deficiency of the monoaminergic neurons in the brainstem poststroke. The monoaminergic input amplifies the small motor neurons’ current non-linearly and causes the slow-twitch motor units to sustain the contraction even after the action potential is over (27). After brainstem stroke, the monoaminergic regulation of the input–output properties might be affected (28, 29), and the contribution of the slow-twitch motor units in the masseter muscles was then lowered.

The fApEn means of all the muscles in the stroke patients were smaller than those in healthy subjects. Previous studies reported that fApEn might be related to the amount of recruited motor units and the firing rate (30). Therefore, the decrease of fApEn in the three muscles might be due to the changes in the neurological control after stroke, such as reduction of the number of recruited motor unit or lowering of the firing rate. The decreased firing rate in stroke patients was recently proven by a high-density EMG study (31). The large variability of fApEn in stroke patients suggested that large arithmetic cancels should happen when calculating means, hence some real situation might be hidden behind the fApEn means. In the case-by-case plot (Figure 5C), Patient 3 and 6 showed marked lower fApEn values of the left masseter muscle, while the other patients’ data lied around the healthy subject’s fApEn mean.

### Influences of Muscles

For healthy subjects, the right masseter muscle yielded a higher RMS compared to the left one, which could be interpreted by different activations of the working-side and the balancing-side of the masseter muscles (32). It might reflect the truth that most (13/15) of the healthy subjects in this study habitually chewed with their right side. However, we observed no significantly different MDF and fApEn among different muscles. It might suggest that the neurological control of the jaw muscles in healthy subjects may be homologous.

For patients, mean values of the RMS and MDF between bilateral masseter muscles were statistically different, and the bilateral temporal muscles exhibited significantly different RMS but non-significantly different MDF. The lower RMS means of the right masticatory muscles might be due to the weakness of the right masticatory muscles. According to the case-by-case RMS plot, four patients exhibited right-side weakness, but only two of them had right face sagging (Table 1). EMG analysis might reveal the unseen weakness of the patients. MDF is a parameter often used to characterize fatigue (33), and both masseter and temporal muscles yield lower MDFs in a sustain clench fatigue experiment (34). Previous studies have reported that successive recruitment of new motor units might be the reason of spectrum shift during muscular fatigue (35). The task in our study was fatigue-free, and the higher MDF in the right masseter muscle might due to the smaller action duration of the motor units (36).

The large variability of the fApEn implied that it might be more sensitive to the pathological status of the patients. From the case-by-case fApEn plot, we observed that most of the data points of patients were lower than the corresponding healthy subjects’ means, which was in agreement with previous study showing smaller fApEn in the patient group (37). Combined fApEn with RMS, we might obtain more information about the masticatory function of the patient. For example, although Patient 5 and 6 had Type II RMS muscle pattern, the right masseter muscle’s data of Patient 5 lied within the mean ± SD interval of healthy subjects, while Patient 6’s data lied markedly beneath that interval. It suggested a larger deficiency of the neuromuscular control of the right side in Patient 6, and this patient had a form of right face sagging (Table 1).

### Influences of Head Positions

Changing head position is a kind of static vestibular stimulation (5), which interferences balance control. In the other hand, occlusion is widely interrelated to gaze stabilization (38) and balance control (39) through activating complex nervous reflexes (40). A previous study (4) observed two types of EMG responses of the masticatory muscles to changes in head position. In the balanced type, the corresponding bilateral muscles (e.g., left and right temporal muscles) were activated equally when subjects performing ventral and dorsal flexion, while ipsilateral activation was observed in the rotation or tilting of the head. The unbalanced type exhibited asymmetrical and irregular activations. Although our results showed no statistical significance of head position, there exhibited similar trends to Ref. (4), i.e., the right muscles of the healthy subjects were much more activated when rotating to the right. The neuromuscular mechanism of changes in head position influencing muscle activation might be due to the tonic neck reflex, which was demonstrated with rats (41). The reasons why head position was a non-significant effect in our study might be due to the interindividual variation, also pointed out in the previous study (4), and the different experimental protocol we used, i.e., we instructed the subjects to clench as hard as possible instead of recording their rest EMG signals. Biting hard activated the whole muscle synchronously (42), and the weak tonic-neck-reflex origin EMG signal would be buried in the large firing signal. However, the sternocleidomastoid muscles would be co-activated (43) during maximum voluntary clenching. Therefore, the tonic neck reflex might be actually enhanced (44) although we could not see it. From the case-by-case EMG parameter values to head position curve of patients (data not show), we found again the high heterogeneity of patients’ performance, but the clinical relevance was not clear.

### Limitation of the Study

In the current study, we have investigated the muscle activities in response to variations in head position using three EMG parameters in both the healthy subjects and brainstem stroke patients. However, several limitations still should be addressed. First, we recruited the healthy youths rather than age-matched healthy adults. It should be noted that age might be one of the factors that influenced the EMG parameters, but age effect was not included in this study. In this preliminary study, we mainly focused on whether the three EMG parameters were discriminating and suited for assessing the masticatory function poststroke. The age effect should be further studied in the future. Second, more patients should be recruited in the future study to improve the statistical power of the study.

## Conclusion

In this study, three EMG parameters, RMS, MDF and fApEn, were used to evaluate the activities of bilateral masseter and temporal muscles at different head positions in healthy subjects and brainstem stoke patients. We found that subject and muscle effected significantly on these parameters, but head position was a non-significant effect. The stroke patient group performed differently during MVC compared with healthy subjects and exhibited a large heterogeneity. Several patterns and trends were detected using multiparameter EMG analysis. Multiparameter EMG analysis might provide rich information and should be a potential useful tool of quantifying the neural activity related movement patterns of the deficient masticatory muscles poststroke.

## Ethics Statement

This study received permission from local ethics committee. All the participants were volunteered to take part in this study and signed the written informed consent about the purpose and procedures of the study prior to the experiment.

## Author Contributions

Both CJ and MW conducted most of the experiments, collected and analyzed the data, interpreted the results, and finished the draft manuscript. JL designed the study, helped to analyze the data, and interpreted the results and revised the manuscript. JL and WZ participated in the data collection and analysis. WH conducted part of the experiments, recruited the subjects, collected data, and interpreted the results. RS designed the study and helped to interpret part of the results.

## Conflict of Interest Statement

The authors declare that the research was conducted in the absence of any commercial or financial relationships that could be construed as a potential conflict of interest.
